# Advancing Post-Genome Data and System Integration Through Machine Learning

**DOI:** 10.1002/cfg.129

**Published:** 2002-02

**Authors:** Francisco Azuaje

**Affiliations:** Department of Computer Science University of Dublin – Trinity College Dublin 2 Ireland

## Abstract

Research on biological data integration has traditionally focused on the development of systems for the maintenance and interconnection of databases. In the next few years, public and private biotechnology organisations will expand their actions to promote the creation of a post-genome semantic web. It has commonly been accepted that artificial intelligence and data mining techniques may support the interpretation of huge amounts of integrated data. But at the same time, these research disciplines are contributing to the creation of content markup languages and sophisticated programs able to exploit the constraints and preferences of user domains. This paper discusses a number of issues on intelligent systems for the integration of bioinformatic resources.


**Comparative and Functional Genomics**
*Comp Funct Genom* 2002; **3**: 28–31.
**DOI:** 10.1002/cfg.129

**Conference Review**

### Advancing post-genome data and system integration through machine learning

Francisco Azuaje*
*Department of Computer Science, University of Dublin – Trinity College, Dublin, Ireland*

**Correspondence to:*
*Francisco Azuaje, Department of Computer Science, University of Dublin – Trinity College, Dublin 2, Ireland.*
*E-mail: Francisco.Azuaje@cs.tcd.ie*

Received: 30 October 2001
Accepted: 16 November 2001
Published online:
4 December 2001

### Abstract

**Research on biological data integration has traditionally focused on the development of systems for the maintenance and interconnection of databases. In the next few years, public and private biotechnology organisations will expand their actions to promote the creation of a post-genome semantic web. It has commonly been accepted that artificial intelligence and data mining techniques may support the interpretation of huge amounts of integrated data. But at the same time, these research disciplines are contributing to the creation of content markup languages and sophisticated programs able to exploit the constraints and preferences of user domains. This paper discusses a number of issues on intelligent systems for the integration of bioinformatic resources. Copyright © 2001 John Wiley & Sons, Ltd.**

**Keywords:** intelligent data integration; artificial intelligence; semantic web; post-genome bioinformatics

### Intelligent integration of bioinformatic resources

Research on bioinformatic integration has traditionally focused on: a) the design of standards to represent and maintain biological data and knowledge bases [6]; and b) the development of protocols to interconnect heterogeneous and distributed databases [3]. These and more advanced initiatives will provide the core elements for the creation of a global biological knowledge management infrastructure. This process will not only allow the exchange of information between users, but it will also allow computer programs to automatically search, retrieve and analyse biological content on the web. Furthermore, *agent-based technologies* may become a fundamental knowledge discovery approach [7], in which distributed programs will interact with multiple web resources and humans to improve their cooperation capabilities.

It is well known that artificial intelligence (AI) and data mining techniques are capable of analysing multiples sources of data, which may be acquired from different sensors, represented by different formats and aimed to describe several features of a complex problem domain [2]. But also it has been shown that this knowledge management paradigm may significantly support the birth of the *post-genome semantic web* mentioned above. Important aspects for the creation of such a semantic web include the design of machine-readable web content and reasoning models [5]. AI-inspired content *markup languages* are currently being developed and evaluated in domains such as electronic business [10]. These languages and interactive systems will be based on well-defined semantics and processing rules, which will allow a more effective manipulation of web-based resources. However there are several computational factors that deserve further research.

This paper aims to overview some of the problems currently investigated by the computational intelligence community in order to achieve some of the goals of the semantic web. Similarly, it discusses some of the potential applications to support the development of breakthrough technologies for post-genome data and knowledge integration. This review is not comprehensive due to space constraints and the growing number of concepts under development. We hope that this contribution

Copyright © 2001 John Wiley & Sons, Ltd.

will guide our readers to further discuss these integration challenges.

### A post-genome semantic web: basic requirements and components

Figure 1 illustrates some of the building modules required in the construction of an intelligent integration infrastructure for the post-genome era. Empty boxes represent possible components or goals, which are not included in this review. A fundamental condition that has already been achieved is the existence of multiple sources of distributed and heterogeneous data. This information will need to be represented and stored using standards to facilitate their exchange and analysis. Module (*i*) in Figure 1 includes some of the techniques used to approach this problem, such as *eXtensible Markup Language* (XML), XML schemas or document type definitions, and *Resource Description Framework* (RDF). A bioinformatic service may be defined as a software platform, which is able to provide users with automatic ways to search, retrieve and analyse information. Module (*j*) consists of the implementation of bioinformatic services in a *machine-readable* form. Another fundamental component is illustrated in module (*k*). A well-known problem is the difficulty in finding and using the many bioinformatic databases and services currently available on the web. Therefore *agent technology* has become a promising approach to deal with some of the complexity, reliability and autonomy issues required to support integrative knowledge discovery tasks [8]. The following sections discuss a number of aspects that need to be studied for the development of the platform depicted in Figure 1.

### Three relevant integration problems

### Mining XML data and metadata

XML has become an important choice for data representation and exchange in the biotechnology sector. Moreover a number of XML-based 'standards' have been proposed in different domains, including *Biopolymer Markup Language* (BIOML) (**http://www.bioml.com**), and *MicroArray and Gene-Expression Markup Language* (MAGE-ML) (**http://www.geml.org**). One of the most important challenges in this XML explosion will be to identifiy novel and useful patterns from large document collections. These XML databases significantly differ from traditional data representation systems [4]. Although several data mining and artificial intelligence techniques have been successfully applied to different knowledge discovery domains, new solutions will be required to approach this problem.

One fundamental point is how to measure

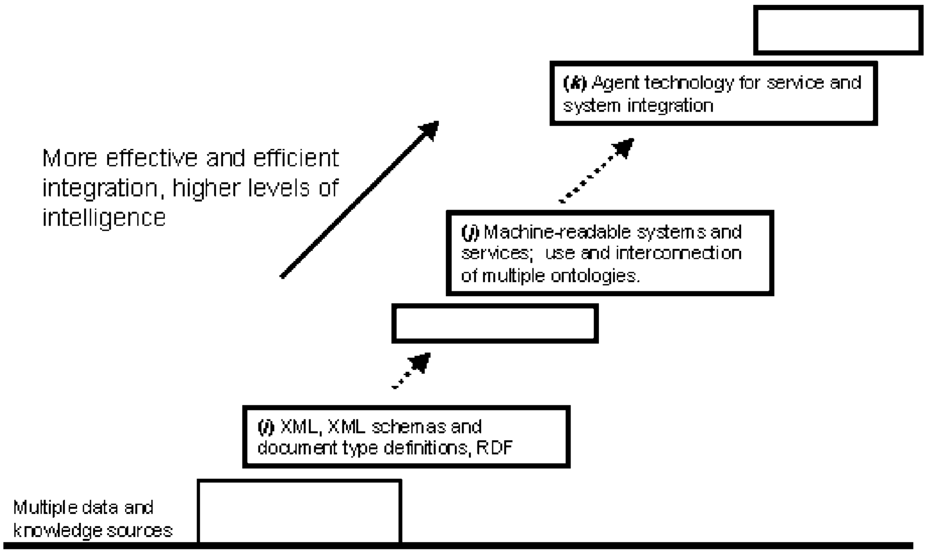

**Figure 1.** Some of the building modules and steps needed to achieve intelligent data and system integration in the post-genome era

Copyright © 2001 John Wiley & Sons, Ltd.

similarity between XML-based data or metadata in order to perform search, retrieval and other recognition tasks. These models should approach similarity at different levels, such as metadata, documents, elements and attributes. This factor is linked to the problem of defining operators for element and attribute comparisons, indexing, and the processing of external references. The data mining research community needs to provide users with inexpensive tools to generate, parse and classify XML resources. Moreover, key data mining functions, such as *clustering* [1], might require new methods due to the multi-layered, structured and heterogenous nature of XML. Data engineers should also propose XML-based methods to represent analysis results. It may allow, for example, multiple data mining systems to share resources and functions, which might represent an effective way to support integration in distributed application environments.

### Automated concept discovery

Effective data and knowledge integration may be achieved only if there is a good understanding of the embedded semantics of the domain under consideration. At the same time it has been suggested that, in spite the heterogeneity of bioinformatic systems, it should be possible to identify a semantic unity within a common application domain. This unity may be expressed, for instance, in the form of similar data structures or usage patterns. It may be represented at various levels such as the database model, data structures and in the knowledge background applied by the community of users. Therefore one of the major objectives for bioinformatics is how to support the discovery of this knowledge.

It has been shown, for instance, that a conceptual integration approach may be based on the discovery of similarities at the metadata level [11]. Such a metadata mining process may be performed on database objects or elements to discover sets of classes. Thus it can provide the basis for a conceptual description of the application domain. The classes or concepts discovered might not only represent fundamental components of the domain ontology, but also they may simplify retrieval and navigation tasks. Figure 2 summarises this concept discovery process based on a metadata clustering algorithm.

### Agent technology for service and system integration

One of the most time-consuming research tasks is to find the right bioinformatic tool or database from the many available on the web. Thus, one useful application is to automate the processes of finding and executing bioinformatic services. An intelligent software agent can be defined as a

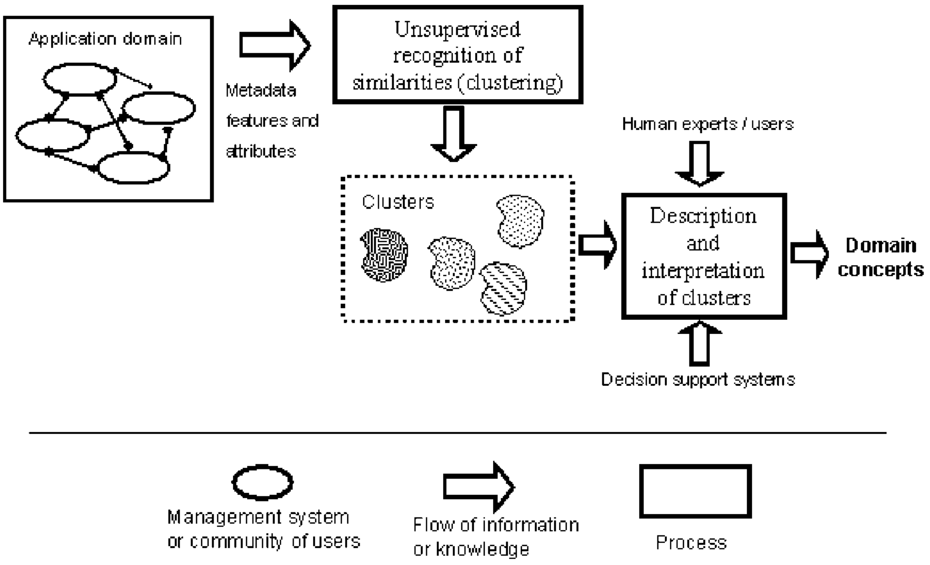

**Figure 2.** A concept discovery framework to support knowledge integration tasks

Copyright © 2001 John Wiley & Sons, Ltd.

computational program that is proactive, autonomous and able to adapt to new situations [7]. It has been suggested that the implementation of agent communities may facilitate Internet-based knowledge discovery tasks [8]. But the success of this story will depend on how we exploit the benefits obtained from using web ontologies.

The semantic markup of web-based systems will represent a knowledge base that will allow agents to exploit users' constraints and preferences for the automatic discovery, execution and composition of web services [10].

(i) **Automatic service discovery.** It involves the automatic localisation of web tools or applications, based on the properties and preferences specified by a user. A user might request for example: to 'find a site that provides genome expression data originating from normal and aberrant prostate tissues'; or to 'find a *Blast* tool based on the algorithm developed by¥'.
(ii) **Automatic web service execution.** It consists of automatically executing a web-based application. A user might request to *blast* a sequence using a customised set of *parameters* and a tool, which can be found on a specific *URL*; or the user might request to *download* expression data from a particular public database.
(iii) **Automatic composition of web services.** In this task agents should be able to perform complex functions based on the automatic selection and execution of services. These agents should also generate responses to those processes. For example a user might request 'to blast a number of sequences using different tools available on the web'; or the user might request to 'classify expression samples using the most robust clustering algorithms available for this type of data'.

Unfortunately none of these applications are entirely available today due to the limitations of the existing web. For instance there is the need to improve the capabilities of markup languages. Recent efforts include the *DARPA Agent Markup Language* (DAML) initiative, which focuses on the development of declarative representations of web services, domain constraints and user preferences [9]. DAML is an AI-inspired model built on RDF. This type of project provides the basis for representing the data and metadata associated with a web-based service (including constraints and capabilities), the protocols for its execution and the consequences of it use. These factors are crucial to support the development of agent technologies for service and system integration.

### Final remarks

This paper has addressed current research, opportunities and challenges to achieve knowledge integration in the post-genome era. Some of these ideas have already been discussed within the context of other application domains such as e-commerce. Artificial intelligence techniques together with advances in ontologies and semantic markup languages represent a promising approach to simplifying time-consuming research tasks in distributed bioinformatic environments. Furthermore, this synergy may significantly support knowledge discovery processes in biosciences. Finally, this review has also been intended as an invitation to foster new and stronger links between the bioscience and computer science communities.

### References

1. Azuaje FA. 2001. A cluster validity framework for genome expression data. *Bioinformatics (in press)*.
2. Azuaje F, Dubitzky W, Black N, Adamson K. 1999. Improving clinical decision support through case-based fusion. *IEEE Trans Biomed Eng* **46**: 1181–1185.
3. Barillot E, Leser U, Lijnzaad P, *et al*. 1999. A proposal for a standard CORBA interface for genome maps. *Bioinformatics* **15**: 157–169.
4. Bertino E, Catani B. 2001. Integrating XML and databases. *IEEE Internet Computing* **5**: 84–88.
5. Fensel D. 2000. The semantic web and its languages. *IEEE Intelligent Systems* **15**: 67–73.
6. Frishman D, Heumann K, Lesk A, Mewes HW. 1998. Comprehensive, comprehensible, distributed and intelligent databases: current status. *Bioinformatics* **14**: 551–561.
7. Furukawa K, Michie D, Muggleton S. 1999. *Intelligent agents*. Oxford University Press: Oxford; 515.
8. Hendler J. 2001. Agents and the semantic web. *IEEE Intelligent Systems* **16**: 30–37.
9. Hendler J, McGuiness D. 2000. The DARPA agent markup language. *IEEE Intelligent Systems* **15**: 72–73.
10. McIlraith S, Son T, Zeng H. 2001. Semantic web services. *IEEE Intelligent Systems* **16**: 46–53.
11. Srinivasan U, Ngu A, Gedeon T. 2000. *J Am Soc Inf Sci* **51**: 707–723.

Copyright © 2001 John Wiley & Sons, Ltd.
